# Tuning Self‐Assembly of Hole‐Selective Monolayers for Reproducible Perovskite/Silicon Tandem Solar Cells

**DOI:** 10.1002/smtd.202401758

**Published:** 2025-02-25

**Authors:** Oussama Er‐Raji, Stefan Lange, Carl Eric Hartwig, Adi Prasetio, Martin Bivour, Martin Hermle, Marko Turek, Stefaan De Wolf, Stefan W. Glunz, Juliane Borchert, Patricia S. C. Schulze

**Affiliations:** ^1^ Fraunhofer Institute for Solar Energy Systems ISE Heidenhofstr. 2 79110 Freiburg Germany; ^2^ Chair of Photovoltaic Energy Conversion Department of Sustainable Systems Engineering (INATECH) University of Freiburg Emmy‐Noether‐Str.2 79110 Freiburg Germany; ^3^ Fraunhofer Center for Silicon Photovoltaics CSP Otto‐Eissfeldt‐Str. 12 06120 Halle Germany; ^4^ KAUST Solar Center (KSC) Physical Sciences and Engineering Division (PSE) King Abdullah University of Science and Technology (KAUST) Thuwal 23955 Saudi Arabia

**Keywords:** hole transport layers, perovskite silicon tandem solar cells, Photovoltaics, reproducibility, self‐assembled monolayers

## Abstract

Self‐assemble monolayers (SAMs) have become state‐of‐the‐art hole‐selective contacts for high‐efficiency perovskite‐based solar cells due to their easy processing, passivation capability, and low parasitic absorption. Nevertheless, for the deposition of SAMs with a monolayer thickness and a high packing density on metal oxide substrates, critical challenges persist. To overcome these, the study focuses on the impact of annealing temperature – an intrinsic yet so far unexplored process parameter – during the formation of SAMs. By performing in situ angle‐resolved X‐ray photoelectron spectroscopy combined with advanced data analysis routines, it is revealed that increasing the annealing temperature reduces the formed SAM layer thickness from a multilayer stack of ≈5 nm at 100 °C (conventional temperature employed in literature) to a monolayer at 150 °C. Furthermore, denser adsorption of the SAM to the metal oxide surface is promoted at high temperatures, which enhances the interfacial SAM/perovskite passivation quality. With this strategy, a 1.3%_abs_ power conversion efficiency (*PCE*) increment is obtained in fully‐textured perovskite/silicon tandem solar cells, with improved reproducibility, and a champion device approaching 30% *PCE*. This study advances the understanding of SAMs formation and presents a promising strategy for further progress in high‐efficiency perovskite‐based solar cells.

## Graphical abstract

This study investigates how annealing temperature affects the formation of hole‐selective self‐assembled monolayers. Advanced in situ X‐ray photoelectron spectroscopy uncovers that increasing the annealing temperature beyond the conventional 100°C reduces the thickness of 2PACz from a multilayer stack of ∼5 nm to a monolayer at 150°C and improves its packing density. Consequently, fully‐textured perovskite silicon tandem solar cells achieve ∼30% *PCE*.

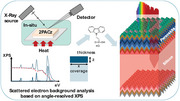

## 1 Introduction

Presently, perovskite solar cells and perovskite/silicon tandem solar cells have reached the desired power conversion efficiencies (*PCE*s) to be considered true contenders for wide‐scale photovoltaic deployment.^[^ ^1^, ^2^, ^3^, ^4^ ^]^ Among the different material developments that made that possible is the emergence of self‐assembled monolayers (SAMs) as hole‐selective contacts.^[^ ^5^, ^6^ ^]^ SAMs typically consist of three components: an anchoring group, a spacer group, and a functional group. By tailoring the different components via chemistry design, SAMs can improve the solar cell performance through tuning energy‐level alignment for faster charge extraction,^[^ ^7^, ^8^, ^9^ ^]^ modulating the substrate's surface energy for enhanced perovskite crystallization,^[^ ^10^, ^11^, ^12^, ^13^ ^]^ and passivating the buried interface of the polycrystalline perovskite film.^[^ ^14^, ^15^, ^16^, ^17^, ^18^ ^]^ Furthermore, they have been demonstrated to form both via solution processing and thermal evaporation which renders their scalability possible.^[^ ^19^, ^20^, ^21^, ^22^ ^]^

A densely packed SAM with minimal defects and a monolayer formation is crucial for high‐efficiency and reproducible solar cells. However, owing to the nature of the employed processing methods (e.g., spin coating, evaporation) it is difficult to achieve these objectives. Typically, SAMs tend to aggregate or crystallize, driven by weak Coulomb forces and strong Van der Waals interactions, particularly π–π interactions.^[^ ^2^, ^15^, ^23^, ^24^, ^25^, ^26^, ^27^, ^28^ ^]^ The use of alcoholic processing solvents, due to their amphiphilic nature, further promotes this tendency.^[^ ^29^ ^]^ On the other hand, thermally evaporated SAMs yield several nanometers thick layers with different orientations on the metal oxide substrate.^[^ ^19^ ^]^ This results in a deviation of the layer's overall dipole and jeopardizes the *PCE* at the device level.

To solve the problem of monolayer formation, a post‐washing step after the annealing treatment is standardly employed and fulfills the removal of excessive non‐covalently bonded molecules.^[^ ^10^, ^11^ ^]^ Besides, literature reports have demonstrated that tailoring the crystallinity and the thickness of the underlying metal oxide can tackle the issue of packing density enhancement.^[^ ^3^, ^30^, ^31^, ^32^ ^]^ Other reports focused on tuning the SAM solution components by adding adjacent molecules or tuning the solvent system, which prevents aggregation, and thus enhances the packing density and molecular orientation of the initially formed monolayer.^[^ ^2^, ^15^, ^23^, ^24^, ^25^, ^26^, ^27^, ^28^ ^]^

Nevertheless, to date, the post‐annealing temperature of SAMs has not been considered for fulfilling both requirements of monolayer formation and packing density for application in perovskite solar cells. The standardly used temperature in the community is 100 °C, which dates back to the first report by Al Ashouri et al. in 2019.^[^ ^33^, ^34^ ^]^ Generally, the annealing strategy is a critical parameter that has been shown to regulate molecular ordering, charge carrier mobility, as well as impact the thickness of organic layers in different optoelectronic fields.^[^ ^35^, ^36^, ^37^, ^38^ ^]^

Inspired by this, in this work, we study the impact of annealing temperature on the packing density and monolayer formation of a popular SAM, namely 2‐(9H‐carbazol‐9‐yl)ethyl)phosphonic acid (2PACz). Through a series of characterizations, we find that increasing the annealing temperature above the conventional 100 °C enhances the passivation quality at the SAM/perovskite interface. By employing in situ angle‐resolved X‐ray photoelectron spectroscopy and scattered electron background analysis, we reveal that increasing the annealing temperature particularly reduces the thickness of the formed SAM from a multilayer of 4–5 nm at 100 °C to a monolayer of ≈1 nm at 150 °C. Furthermore, the analysis of the full spectrum reveals a stronger interaction between the SAM's anchoring group and the underlying metal oxide at high temperature, thus indicating enhanced packing density. With this strategy, fully‐textured perovskite/silicon tandem solar cells with optimized annealing temperature demonstrate *PCE*s approaching 30%. Additionally, an improved reproducibility is observed. This study advances the understanding of SAMs formation and proposes a simple yet effective strategy for further performance enhancement of single‐junction and perovskite‐based tandem devices.

## 2 Results and Discussion

### 2.1 Impact of SAM Annealing Temperature on Its Properties

**Figure 1:**
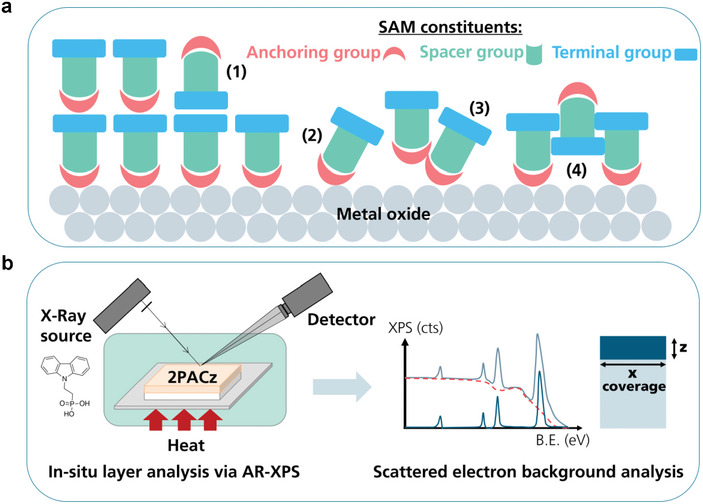
Schematics of potential defects in self‐assembled monolayers (SAMs) and characterization method for assessment. a) Potential defects in self‐assembled monolayers deposited on a metal oxide substrate: (1) molecules where the anchoring group is not covalently bounded to the substrate and thus adsorb on the formed monolayer leading to the formation of a “multi”‐layer stack of SAMs instead of a monolayer, (2) tilted or misaligned molecules, (3) aggregated molecules leading to the formation of dimers trimers etc., and (4) voids created by molecules’ interaction on the substrate which lead to non‐full coverage (adopted from Hinckley et al.).^[^ ^39^ ^]^ b) Schematic depicting the employed in situ angle‐resolved X‐ray photoelectron spectroscopy (AR‐XPS) characterization method and the data evaluation routine to investigate the formation of SAMs.

We investigated the impact of annealing temperature on the formation of self‐assembled monolayers using 2PACz as the SAM molecule and a mixed‐cation, mixed‐anion perovskite thin film. The SAM was formed via spin coating, followed by a 10 min annealing at the specified temperature, without additional washing. **Figure** **1**a depicts the challenges during SAM formation on a metal oxide substrate and Figure 1b portrays the main characterization tool for assessment of the layer quality.

**Figure 2:**
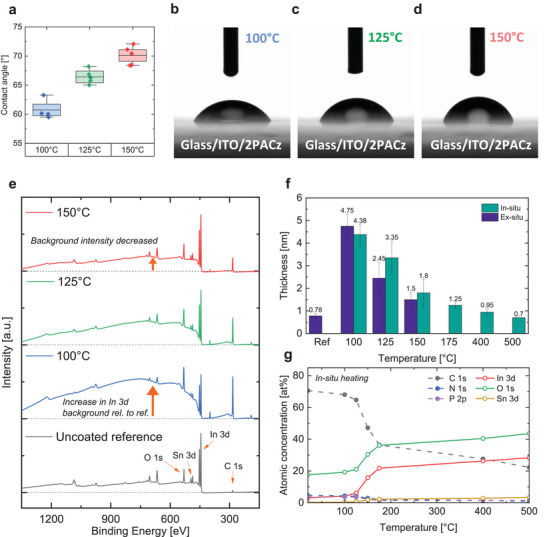
Impact of 2PACz annealing temperature on the formed layer's properties. a) Contact angle measurements of glass/ITO/2PACz substrates with a variation in 2PACz annealing temperature and images of a representative water droplet at b) 100 °C, c) 125 °C, and d) 150 °C. e) X‐ray photoelectron spectroscopy (XPS) survey of an uncoated glass/ITO substrate reference, and glass/ITO/2PACz samples after ex situ annealing at 100, 125, and 150 °C. f) Fitted 2PACz thickness using scattered electron background analysis on insitu annealed samples (in green) and comparison to ex situ annealed samples (in purple). g) Atomic composition change as a function of 2PACz annealing temperature (in situ, calculated assuming a homogeneous medium). With increasing annealing temperature, the thickness of the 2PACz layer decreases.

For a qualitative evaluation of changes in surface energy, we measured the contact angle formed between droplets of water and glass/ITO/2PACz stacks (**Figure** **2**a–d). The increase of annealing temperature from 100 to 150 °C led to a systematic average increase in contact angle from 61° to 70°. The ≈15% increase in contact angle highlights an increased hydrophobicity at high annealing temperature which has been previously ascribed to either 1, reduction/removal of hydrophilic phosphonic acid groups on the surface of the last monolayer (i.e., misoriented 2PACz molecules) and/or 2, an enhanced packing density of the formed SAM (i.e., better coverage/reduced voids where water would directly contact the ITO substrate).

For a better understanding of the changes happening at the molecular level, in situ, angle‐resolved X‐ray photoelectron spectroscopy (XPS) measurements were carried out (Figure 1b). A sample consisting of glass/ITO/2PACz, where 2PACz was spin coated and annealed at 100 °C for 10 mins, was successively heated in situ. We then estimated the change in 2PACz thickness and coverage using scattered electron background analysis. The principal methodology for data evaluation consisted of using the inelastic photoelectron background. After excitation, inelastic scattering processes of photoelectrons during the way to the sample surface result in a distinct background shape at the low kinetic energy side of (elastic) XPS peaks. Both the sample nanostructure (conformal overlayer, buried layer, coated substrate, homogeneous material, or nanoparticles on a substrate) and the material itself influence the XPS background shape and magnitude. This method becomes valuable when different nanostructures lead to the same elastic peak intensities, and only a change in the background signal can enable their differentiation. Practically, the background signal is simulated based on a nanostructural sample model, and it is compared to the measured background shape. A series of iteration is done until convergence toward the measured data is achieved.

The XPS survey of the uncoated ITO reference sample featured In, O, and Sn peaks originating from ITO (Figure 2e). Moreover, a small C 1s peak was present due to carbon contamination from the atmosphere during sample preparation. As shown in Figure 2e, the inelastically scattered background left of the In 3d peak was relatively flat. With the addition of a 2PACz layer annealed at 100 °C, the C 1s became more prominent and the background of the In 3d peak increased significantly in intensity in comparison to the reference, forming a rock‐like structure. As the annealing temperature progressively increased above 100 °C and until 150 °C, this structure faded out to almost the same background shape as that of the bare ITO sample.

By modelling the surface nanostructure and comparing it with the experimental results, we found that coverages in the range of 90–100% fit best the measured background shape of the In 3d peak for all temperature variations. The fitted overlayer thicknesses – here overlayer designates the stack on top of ITO as it could be a combination of 2PACz as well as surface adsorbates – are shown in Figure 2f. At 100 °C, the estimated thickness was in the range of 4–5 nm. With increasing 2PACz annealing temperature the thickness decreased reaching ≈2 nm at 125 °C and ≈1 nm at 150 °C, which is the expected thickness for a 2PACz monolayer.^[^ ^40^ ^]^ After that, the change in thickness was minimal and the thickness beyond 400 °C was found to be comparable to that of the uncoated sample, which hints toward the near‐complete removal of 2PACz.

To verify the reproducibility of these results a set of glass/ITO samples with 2PACz annealed at distinct temperatures (i.e., ex situ heating at 100, 125, and 150 °C for 10 mins) were measured and yielded similar quantitative results as highlighted in Figure 2f.

Besides background shape analysis, we further examined the changes in atomic composition. Figure 2g shows that the largest change occurred in the 100–175 °C temperature range, where a significant decrease in C was noted (68% to 37%). Similarly, the N and P signals exhibited a slight 2%_abs_ decrease. Simultaneously, a notable increase in O and In was observed (20% to 38%, and 5% to 22%, respectively), and to a lesser extent a rise in Sn was noted (2.5%_abs_). These changes indicate that increasing the annealing temperature reduces the amount of detectable 2PACz elements on the substrate and favors the detection of the underlying ITO layer elements, which agrees with the determined thinning down of 2PACz thickness with temperature (Figure 2f), consistent with contact angle data (Figure 2a).

**Figure 3:**
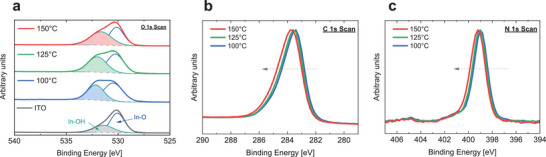
Understanding the impact of increased 2PACz annealing temperature through analysis of the detailed XPS spectrum. Detailed XPS spectrum of the a) O 1s, b) C 1s and c) N 1s of ex situ annealed 2PACz/ITO samples with increasing annealing temperature. With higher temperature, a reduced P─O contribution to the O 1s peak is observed together with a shift of C 1s and N 1s peaks to higher energies.

An inspection of XPS detailed spectra revealed further modifications of the SAM‐coated ITO surface (**Figure** **3**). First, the O 1s peak of the uncoated ITO reference sample exhibited an asymmetric peak shape, attributable to a main peak at 530.1 eV (In─O bonds) and a secondary peak at 531.4 eV, which is caused by surface hydroxide bonds (In─OH) or In in an oxygen‐deficient surrounding.^[^ ^41^, ^42^ ^]^ Coating 2PACz on the ITO substrate led to the appearance of a distinct O 1s component due to P─O bonds originating from the phosphonic acid (PA) group at ≈532.2 eV binding energy besides the In─O main peak. The formerly mentioned peak at 531.4 eV cannot be separated anymore from the P─O component due to energetic overlap and peak widths. As the annealing temperature increased, the intensity of the PA component decreased relative to ITO main component, indicating removal of excess PA. Furthermore, an energetic shift of SAM and ITO‐related peak positions to higher binding energies was observable with increasing annealing temperatures (Figure 3b,c). This indicates either an increase of work function (assuming the Fermi level relative to the valence band is fixed) or a Fermi level shift away from the valence band (assuming a constant work function).

In a second step, we applied a similar methodology to textured Si substrates (random pyramids with a range of 1–4 µm, Figure S1, Supporting Information)^[^ ^43^ ^]^ for a coaxial setup (macroscopic surface normal parallel to electron analyzer axis) assuming highly symmetric silicon pyramids, all with the same base angle of θ = 55°. This mimics the geometry of an angle‐dependent XPS measurement, where the sample is tilted and the angle between the analyzer and the surface is 90° − θ = 35°. As such, surficial XPS signals of 2PACz on a textured sample are enhanced by a factor of ≈1/sin 35° in comparison to 2PACz on a flat Si substrate with equal nominal thickness. We took this geometry in our simulation model into account to calculate the 2PACz thickness on ITO‐coated textured silicon in dependence on the annealing temperature.

**Figure 4:**
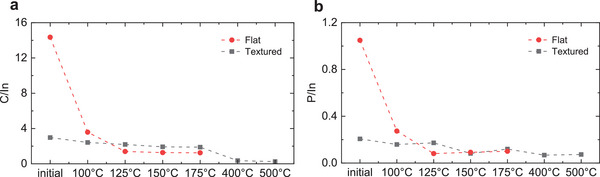
Comparison between flat and textured substrates. Change in a) carbon to indium (C/In) and b) phosphorus to indium (P/In) as a function of annealing temperature, using a sample structure consisting of silicon substrate/ITO/2PACz, with the substrate being flat (red, circle symbols) versus textured (gray, square symbols). The heating was done in situ. A higher impact of 2PACz annealing temperature can be observed on flat substrates.

We found the best fits for 1.0–1.1 nm thickness (error estimated to be <0.5 nm) for coverages between 90% and 100% in the temperature range of 100–150 °C. This indicates that textured substrates initially accommodate a lower density of 2PACz molecules compared to planar substrates. By extending the study to 500 °C via in situ heating, a noticeable drop appeared after 175 °C in both C/In and P/In signals (**Figure** **4**), ascribed to 2PACz elements removal. Figure S3 and Table S1 (Supporting Information) show a comparison of in situ versus ex situ annealed textured samples which further confirms the lower impact observed on this type of substrates. This result might also explain the tendency to use a higher concentration of SAM solution in high‐efficiency perovskite/silicon tandem solar cells based on textured substrates in comparison to flat‐front silicon substrates.^[^ ^3^, ^34^, ^44^, ^45^, ^46^, ^47^ ^]^

Overall, the conducted measurements point out that at the conventional 100 °C annealing temperature, 2PACz is not present as a monolayer, but rather as a multilayer stack, which conforms with recent literature. By increasing the annealing temperature, the loosely bounded molecules are removed, resulting in a monolayer determined at ≈150 °C. It is a similar effect to that of the standardly reported post‐washing step, where non‐covalently bonded self‐assembled molecules are washed off the first monolayer. Nevertheless, in the framework of this study, thermal evaporation is the most probable mechanism behind the reduced 2PACz thickness.

### 2.2 Impact of SAM Annealing Temperature on Perovskite Properties and Perovskite/Silicon Tandem Solar Cell Performance

**Figure 5:**
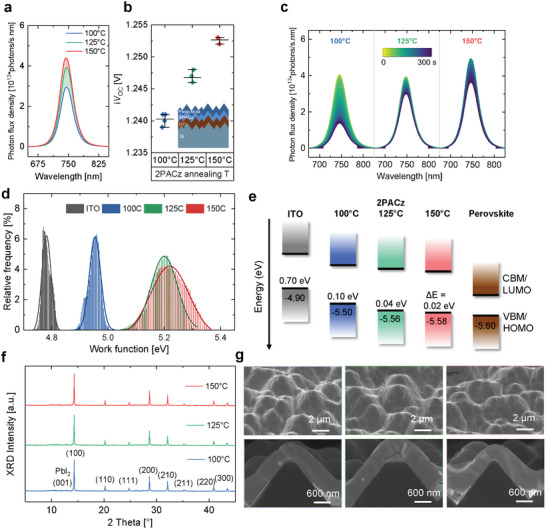
Impact of 2PACz annealing temperature on perovskite and 2PACz/perovskite interfacial properties. a) Absolute photoluminescence (PL) response, b) implied open‐circuit voltage (i*V*_OC_) determined from *PLQY* analysis, and c) PL response tracked under 1‐sun over 300 s in the air (unencapsulated samples) of stacks based on textured silicon/ITO/2PACz/perovskite with a variation in 2PACz annealing temperature. d) Work function of 2PACz‐modified ITO substrates annealed at different temperatures compared to the ITO reference (from KPFM measurements). f) Energy band offset (∆E) between the HOMO level of 2PACz‐modified ITO substrates annealed at different temperatures and the VBM of the perovskite (from PESA measurements). f) X‐ray diffraction pattern and g) morphology (top‐view and cross‐sectional SEM images) of stacks based on textured silicon/ITO/2PACz/perovskite with a variation in 2PACz annealing temperature. Increased SAM annealing temperature results in enhanced i*V*_OC_, improved perovskite photostability, and better energetic alignment between 2PACz and perovskite, while no changes are observed in the structural and morphological properties of the perovskite.

To assess the impact of SAM annealing temperature on the SAM/perovskite interfacial quality, a mixed‐cation, mixed‐anion perovskite absorber with a composition of FA_0.86_Cs_0.14_Pb(I_0.78_Br_0.22_)_3_ (FA: formamidinium) and bandgap of 1.66 eV is adopted, for its suitability to tandem application. It is processed on industry‐compatible textured silicon with random pyramids (pyramid size between 1 and 4 µm) via the hybrid method,^[^ ^48^ ^]^ which consists of first evaporating a CsI/PbI_2_ scaffold (SEM images in Figure S2, Supporting Information) followed by spin coating an FAI/FABr organohalide solution, with urea as a crystallization agent, and annealing.^[^ ^49^, ^50^ ^]^ We note that, as the annealing of the perovskite absorber is done at 100 °C, it allows a direct assessment of the impact of the 2PACz annealing temperature (no exposure of 2PACz to higher annealing temperatures during perovskite film formation). Further details are in the experimental section for a description of the full‐device fabrication protocol. Optoelectronic, structural, and morphological characterization appear in **Figure** **5**.

To characterize the impact of the annealing temperature on the interfacial passivation quality, we conducted spectrally‐resolved steady‐state photoluminescence (ss‐PL) measurements by exciting perovskite thin films in the half‐stack textured silicon/ITO/2PACz/perovskite at an excitation fluence that is relevant for device operation (1‐sun equivalent photon flux). By increasing the temperature from 100 to 150 °C, the perovskite PL's response exhibited an increased PL peak intensity without a change in the PL peak position (Figure 5a). Using PL quantum yield (*PLQY*) analysis, this change corresponded to a ≈13 mV increase in the i*V*_OC_ of the half‐stack (Figure 5b), thus reflecting a reduction of non‐radiative recombination for high‐temperature annealed 2PACz half‐stacks. More importantly, by tracking the evolution of the PL signal of unencapsulated half‐stacks over a period of 300 s in air, a more stable PL peak intensity was found with the 125 and 150 °C variations compared to the reference (Figure 5c; Figure S4, Supporting Information). Extending the optoelectronic study, we performed transient PL (trPL) measurements to gain additional information about charge carrier dynamics. The fitted charge carrier lifetime increased from 893 ns at 100 °C to 1041 ns at 125 °C, and further to 1075 ns at 150 °C (Figure S5, Supporting Information). These improvements confirm a reduction of the defect density in high‐temperature annealed 2PACz half‐stacks, originating from reduced non‐radiative recombination either at 1) the 2PACz/perovskite interface, or 2) the perovskite bulk (e.g., due to a change of substrate‐dependent perovskite growth).

X‐ray diffraction (XRD) and scanning electron microscopy (SEM) measurements were carried out to explore whether the enhanced perovskite optoelectronic properties originate from an improved morphological/structural absorber quality at elevated 2PACz annealing temperature (e.g., due to a different substrate‐dependent growth mechanism). Figure 5f shows no change in the diffraction pattern (no shift of XRD peaks, change in preferential orientation, nor emergence of new phases). Similarly, cross‐section and top‐view SEM images did not show a significant change in the perovskite morphology (Figure 5g). We therefore conclude that the optoelectronic enhancement originates from reduced interfacial recombination at the 2PACz/perovskite junction at elevated annealing temperatures of the SAM.

We then assessed the influence of 2PACz annealing temperature on the energetic alignment at the interface with the absorber. For hole‐selective SAMs, a stronger dipole moment results in a deeper work function (W_f_), which is typically advantageous for perovskite solar cells.^[^ ^51^, ^52^, ^53^ ^]^ The dipole strength can be enhanced via 1) increasing the number of aligned SAM molecules, or 2) orderly organizing the SAM molecular orientation with the anchoring group (phosphonic acid) facing the ITO substrate and the functional group (carbazole moiety) facing the perovskite absorber. By applying the high‐temperature annealing strategy of 2PACz above the conventional 100 °C, we anticipate that these effects combine as excessive non‐covalently bonded SAMs are removed (weakening the dipole moment), but the remaining monolayer becomes more ordered (strengthening the dipole moment). To quantify the impact of these factors and assess the change in energetic alignment at the 2PACz/perovskite interface, we carried out photoemission spectroscopy in air (PESA) and Kelvin probe force microscopy (KPFM).

We found that the work function of ITO (4.78 eV) increased to 4.95 eV upon coating 2PACz and annealing at 100 °C (Figure 5d), consistent with previous reports.^[^ ^23^, ^33^, ^54^ ^]^ By increasing the annealing temperature to 125 °C, a large increase was observed in the W_f_ (+0.25 eV) reaching 5.20 eV, and further increasing to 5.22 eV at 150 °C (these results are supported by Kelvin probe measurements with a larger probing scale resolution, as shown in Figure S6, Supporting Information). Deeper values of the W_f_ obtained at elevated temperature, reflect a stronger 2PACz dipole, confirming the larger contribution of improvement in packing density and order of the first formed monolayer, consistent with XPS results (Figure 3). With that, we found that the energetic offset between the highest occupied molecular orbital of 2PACz and the perovskite's valence band maximum (∆E) was reduced from 0.10 eV at 100 °C, to 0.04 eV at 125 °C, and further to 0.02 eV at 150 °C, allowing for a more efficient hole extraction (Figure 5e; Figure S7, Supporting Information). Combined with the reduction in interfacial recombination as determined via ss‐PL and tr‐PL, these effects anticipate an enhancement in the *V*_OC_ and *FF* of devices with high temperature‐annealed 2PACz.

**Figure 6:**
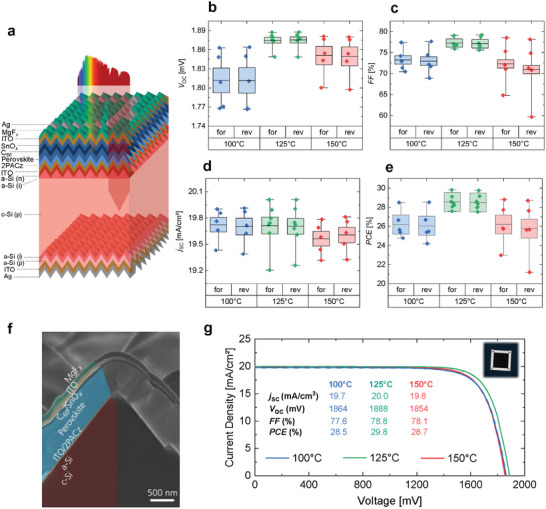
Impact of 2PACz annealing temperature on perovskite/silicon tandem solar cell performance. a) Schematic of the fully‐textured perovskite/silicon tandem solar cell structure studied. Photovoltaic parameters presented in a box plot showing the b) open‐circuit voltage (*V*_OC_), c) fill factor (*FF*), d) short‐circuit current density (*j*_SC_), and e) power conversion efficiency (*PCE*) of the tandem solar cells as a function of 2PACz annealing temperature both in the forward and reverse scans. f) Cross‐sectional SEM image of a representative tandem solar cell. g) Current–density voltage (*J–V*) curves of the best devices (forward in continuous line, reverse in dashed line) and a photograph of a representative solar cell with a 1 cm^2^ active area. Increasing the annealing temperature of 2PACz from 100 to 125 °C improves the performance of the tandem solar cells.

Fully‐textured perovskite/silicon tandem solar cells were fabricated to assess the impact of 2PACz annealing temperature on the device performance (**Figure** **6**a). For our system, an optimum performance was found at 125 °C (Figure 6e).^[^ ^50^ ^]^ More in detail, annealing 2PACz at 125 °C improved the *V*_OC_ (median of 1875 mV) compared to 100 °C (median of 1810 mV), mainly due to enhanced reproducibility. A smaller difference of ≈20 mV in champion devices was noted, which agrees with the i*V*_OC_ measurements (Figure 6b). Furthermore, the *FF* was enhanced from 73% to 77% (median) and from 77.6% to 78.8% for the best devices (Figure 6c), reflecting the enhanced energetic alignment at the 2PACz/perovskite interface. With no major changes in *j*_SC_, in accordance with the *EQE* and reflectance results (Figure S8, Supporting Information), the optimum 2PACz annealing temperature of 125 °C led to a 1.3%_abs_ improvement when comparing the best device to the reference 100 °C, and thus a champion power conversion efficiency of 29.8% was achieved (Figure 6g; Figure S9, Supporting Information). This underlines the robustness of 2PACz formation at higher annealing temperature, contributing to enhanced device performance.

We note that the optimum 2PACz annealing temperature of 125 °C in tandem solar cells differs from the value of 150 °C determined through film characterization, which we attribute to the use of a textured silicon bottom solar cell. As illustrated in Figure 4, the use of textured silicon reduces the impact of the annealing temperature of 2PACz due to the initially lower thickness of the accommodated SAM. Therefore, we anticipate that planar architectures such as perovskite/silicon tandem solar cells with flat‐front silicon or single‐junction perovskite solar cells, will exhibit higher optimal temperatures, approaching 150 °C.

## 3 Conclusion

In summary, the study proposes a simple method to regulate the self‐assembly of hole‐selective monolayers. Through a combination of in situ AR‐XPS measurements and advanced data analysis routines, a correlation was found between the employed annealing temperature and the thickness of the formed hole‐selective layer. In particular, increasing the annealing temperature above the conventional employed 100 °C led to the formation of a robust and compact monolayer without any additional post‐treatment. Owing to the improved packing density of the SAM at high annealing temperature, the SAM/perovskite interfacial passivation quality was enhanced, which led to the fabrication of perovskite/silicon tandem solar cells approaching 30% *PCE*. Our proposed strategy could be beneficial for large area devices, and the findings of the study pave the way for further enhancements in the efficiency of perovskite‐based solar cells.

## 4 Experimental Section

### Perovskite/Silicon Tandem Solar Cell Fabrication—*Silicon Bottom‐Cell Fabrication*

Silicon solar cells were fabricated from 250 µm thick p‐doped silicon wafers (float zone) with 1 Ω cm base resistivity (from Siltronic). A pyramidal texture was etched on both sides of the wafer yielding a pyramid size distribution ranging from 1 to 4 µm, using a KOH aqueous solution (SINGULUS SILEX). After that, the silicon surface was cleaned following an O_3_‐based wet‐chemical cleaning. Then, a stack consisting of intrinsic/doped amorphous silicon passivation layers was deposited on both sides by plasma‐enhanced chemical vapor deposition (PECVD) in an Indeotec cluster tool (powered at 13 560 kHz, at 200 °C using a gas mixture of hydrogen, trimethylboron, silane, and phosphine). The thickness of the intrinsic layers was set to 6 nm and that of the p‐doped and n‐doped layers to 12 nm. Subsequently, the recombination layer was formed through a 1 cm^2^ shadow mask on the front silicon side via DC sputtered ITO (In_2_O_3_/SnO_2_ = 97/3 wt %, VON ARDENNE SCALA in‐line tool) using a mixture of oxygen and argon. Prior to ITO and amorphous silicon deposition, a dipping treatment (aqueous solution of 1% hydrogen fluoride HF) was carried out to eliminate silicon oxide (SiO_2_) from the surface. On the back silicon side, a 195 nm ITO layer was sputtered on the full area. The rear contact was then formed via a 1000 nm thick silver layer. Finally, the 4‐inch wafers were lasered in 7 substrates with 2.5 × 2.5 cm^2^ dimensions, each containing one 1 cm × 1 cm ITO pad (recombination layer) to build one tandem solar cell.

### Perovskite/Silicon Tandem Solar Cell Fabrication—*Perovskite Top‐Cell Fabrication*

The *p*‐*i*‐*n* architecture was adopted for perovskite sub‐cell fabrication. First, 200 µL of ethanol was spin‐coated dynamically (2500 rpm for 30 s) to clean the silicon substrate surface. After that, a UV/ozone treatment was carried out over a 15 min period to eliminate contaminants. The *p*‐contact was based on the self‐assembled monolayer 2PACz (from Dyenamo). The solution was prepared using ethanol as solvent and was pretreated by ultrasonication for 15 mins at room temperature prior to usage. A volume of 100 µL of the solution (4 mm, ethanol as solvent) was statically spin coated (7 s waiting time, 3000 rpm, 30 s) followed by a thermal annealing treatment at a specified temperature (see results and discussion) for 10 mins to ensure the binding of the molecules to the ITO substrate. The SAM deposition was carried out in the glovebox (N_2_‐rich atmosphere). Then, the perovskite absorber was formed via a hybrid evaporation/spin‐coating method. In a Lesker mini Spectros system, CsI (powder from Sigma‐Aldrich) and PbI_2_ (beads from Alfa Aesar) were thermally co‐evaporated from ceramic crucibles to form a 550 nm thick inorganic scaffold. The evaporation rates were set to 0.1 Å s^−1^ (reached at ≈400 °C) and 1 Å s^−1^ (reached at ≈280 °C) for CsI and PbI_2_ respectively and were measured via quartz crystal balances. The substrate temperature was set to 20 °C and the base pressure was <2 × 10^−6^ Torr. Following the formation of the inorganic scaffold, a 150 µL volume of an organohalide solution made by FABr, FAI, and urea (0.67 m solutions of FABr and FAI were prepared in ethanol and added after ≈8 h in a FABr/FAI ratio of 65/35 vol% onto a third vial containing 5 mg mL^−1^ of urea, which was left stirring overnight) was dynamically spin‐coated (2200 rpm, 35 s) followed by an annealing treatment in air at 100 °C for 10 mins (relative humidity of ≈30–55%). The annealing step was carried out in the air to benefit from the positive effect of humidity on perovskite crystallization, as previously described in this work.^[^ ^55^ ^]^ The *n*‐contact was formed using C_60_ (from TCI). The thickness and evaporation rate were set to 21 nm and ≈0.15 Å s^−1^. The top stack was then formed using SnO*_x_* as a buffer layer, ITO as a transparent conductive oxide, Ag as a metal electrode, and MgF*_x_* as an anti‐reflective coating. More in detail, 30 nm of SnO*_x_* were deposited via atomic layer deposition at 80 °C, using de‐ionized water (kept at room temperature) and tetrakis(dimethylamino)tin(IV) (TDMASn, kept at 50 °C) as precursors; 20 nm of ITO was DC sputtered through a shadow mask; 200 nm of Ag were deposited via thermal evaporation at a rate of 5 Å s^−1^; and 100 nm of MgF*_x_* were deposited via thermal evaporation at a rate of 2 Å s^−1^. Note that for thermally evaporated layers, the thickness values are given for a flat substrate. The measured thickness of the pyramid size is lower due to the increased surface area (texture factor of ≈1.4).

### Characterization—*XPS Measurements*

Measurements of ex situ annealed samples were carried out in a Thermo Fisher Scientific Nexsa G2 photoelectron spectrometer with monochromatized Al Kα excitation at 1486.6 eV (operated at 12 kV, 10 mA) and size of the X‐ray spot in the focus of ≈400 µm. A pass energy (PE) of 200 eV has been chosen to record survey spectra. In situ annealing experiments were performed in a Kratos Axis Ultra DLD photoelectron spectrometer under gas background pressures in the order of 10^−7^ mbar. The set temperature range was normally reached within a few minutes and then maintained at the target level for 10 min. No specific heat ramp was set. The measurements themselves were conducted while the sample slowly cooled down. Monochromatic Al Kα excitation was employed too, with an analysis area of approx. 300 × 700 µm^2^ defined by electrostatical/magnetical lenses and an aperture. Survey spectra were recorded with a PE of 160 eV and detail spectra with PE of 40 eV. Independent of the specifically used instrument, two different positions on each sample have been measured and the mean value of all quantified data has been reported in the results section.

Quantification and peak fitting of spectral data were done in Avantage 6.7 and CasaXPS 2.3.19 using instrument‐specific relative sensitivity factors. Inelastic photoelectron background analysis has been accomplished using QUASES‐Tougaard‐Analyze Ver. 7.501.

### Characterization—*SEM Measurements*

A Schottky emission SEM from Zeiss (model Auriga 60, In lens detector) was used to capture cross‐sectional and top‐view electron microscopy images. The top‐view images were taken with an angle of 45° and the acceleration voltage was set to 5 kV.

### Characterization—*XRD Measurements*

X‐ray diffraction measurements were carried out using a Bruker D8 Advance diffractometer, equipped with a Cu anode at 40 mA/40 kV, and a DIFRAC.EVA analysis software. The step size was set at 0.3° and the time per step at 0.1 s.

### Characterization—*Spectrally Resolved Steady‐State PL Measurements*

A LuQY Pro instrument from Quantum Yield Berlin (QYB) was used to perform steady‐state spectrally‐resolved photoluminescence measurements. A 532 nm laser was used, and the spot size and resolution time were set to 0.1 cm^2^ and 3 s respectively. The equivalent laser intensity was set to 1 sun to mimic real‐world operation (automatically calculated for the selected spot size as well as *j*_SC_ and *EQE* at 532 nm).

### Characterization—*Transient PL Measurements*

The samples were excited with a 515 nm diode laser with a spot size diameter of 7.5 mm. Steady‐state conditions of the charge carriers were maintained by setting the pulse width to 240 ms. The laser's on/off ratio was 10^6^ within 1 ns, with the transient recorded during the off‐time. During the on‐time, the laser power was adjusted to match the short‐circuit current density (*j*_SC_) of a perovskite solar cell under 1‐sun illumination (AM 1.5G). A VIS hybrid photodetector, which is read out using a single‐photon counting device, was used to record the PL. The integration time was set to 300 s.

### Characterization—*KPFM Measurements*

A Digital Instruments Multimode AFM (Veeco Metrology Group) equipped with a Bruker head was employed to perform topographic and SKPM potential mapping. For this purpose, an SCM‐PIT V2 AFM tip was utilized, which was fabricated using an RFESP‐75 AFM probe and coated with a reflective layer of Platinum‐Iridium (PtIr). The tip, made of antimony‐doped silicon, featured a resistivity ranging from 0.01 to 0.025 Ω cm. Its rectangular design had nominal frequency and stiffness values of 75 kHz and 4 N m^−1^, respectively.

### Characterization—*Kelvin Probe Measurements*

A Surface Photovoltage Spectroscopy 040 (SPS040) system from KP Technology was used. The work function of the tip (TipWF) was determined to be ≈4.6 eV. This value was calibrated with high precision using gold (Au) and highly oriented pyrolytic graphite (HOPG) substrates as standard reference materials. This measurement, having a larger scale area, was used to confirm the results from KPFM which has only nm‐scale resolution.

### Characterization—*PESA Measurements*

Photoemission spectroscopy in air was conducted on a Riken AC2 setup. The schematic representation of the energy band diagram in Figure 5e was constructed as follows: the E_VBM_ and HOMO levels of the perovskite absorber and the ITO‐modified substrates with 2PACz at different annealing temperatures were determined via PESA measurements. To position the conduction band minimum (E_CBM_) and lowest unoccupied molecular orbital (LUMO) of the perovskite and the ITO‐modified substrates with 2PACz at different annealing temperatures, the optical bandgap of the materials was added on the E_VBM_ and HOMO levels, respectively (from UV–vis measurements).

### Characterization—*J–V Measurements*

Current density–voltage (*J–V*) measurements were performed using a Wacom solar simulator equipped with halogen and xenon lamps. Prior to the measurements, the spectral response of a cell from each variation was measured and used to adjust the two filtered WPVS reference cells following the procedure described by Meusel et al.^[^ ^56^ ^]^ Using a Keithly 2400 source meter, the jV curves were recorded in the range [−100, 1920 mV] with a 20 mV step width and a 34 mV s^−1^ scan speed in forward and then in reverse directions. Between the single measurement points of the *J–V* curve the solar cell is under open circuit condition. The stage temperature was controlled and set to a nominal 25 °C value. The measurement was carried out in air with a relative humidity varying between 20 and 55%. A shadow mask was used to limit the light exposure area to the 1 cm^2^ cell active area.

### Characterization—*EQE Measurements*

The external quantum efficiency was measured with a setup consisting of a Xenon lamp as the light source which was chopped at 133 Hz, a grating monochromator (to produce single‐wavelength light), a trans‐impedance amplifier (to provide bias voltage during measurements), and a lock‐in amplifier (to detect and enhance the signal/noise ratio). Prior to the measurements, a silicon reference cell was used for *EQE* response calibration. Then, the *EQE* of the tandem solar cells was measured in the range of 300–1200 nm (with 10 nm steps) according to Meusel et al.^[^ ^56^ ^]^ Briefly, for measuring the silicon bottom cell, a selective blue bias light illumination was introduced together with a ≈1.08 V electrical bias. For perovskite top cell measurement, a selective infrared bias light was introduced together with a ∼ 0.68 V electrical bias. Note that the measured *EQE*s are not absolute. Details can be found in.^[^ ^57^ ^]^

### Characterization—*Reflection Measurements*

A LOANA tool from PvTools was used to carry out reflection measurements on the tandem solar cells. The setup consisted of tungsten lamps and a monochromatic light from Xenon. The spot size was 3 mm × 5 mm and the reflectance was measured with a BaSO4‐coated integrating sphere under an 8° tilt.

## Conflict of Interest

The authors declare no conflict of interest.

## Author Contributions

O.E.R. and S.L. contributed equally to this work. O.E. conceived the idea, planned the experiments, fabricated the solar cells, performed *J–V*, spectrally‐resolved PL, contact angle, and XRD measurements, and wrote the manuscript. S.L. and C.E.H. participated in experimental planning, performed the ex situ and in situ XPS measurements, and carried out the data analysis. S.L. participated in writing the manuscript. A.P. carried out PESA, KPFM, and KP measurements. M.B. provided silicon bottom solar cells. S.D.W. provided the facility for PESA, KPFM, and KP measurements as well as insights on the results. M.B., M.T., M.H., S.W.G., and J.B. provided the funding (PrEsto, MaNiTU) as well as deep insight into the understanding of characterization data. P.S.C.S. supervised the project and gave essential suggestions about experimental planning, conceptualization, and structure of the manuscript. All authors reviewed the manuscript and repeatedly helped with manuscript reviews.

## Supporting information



Supporting Information

## Data Availability

The data that support the findings of this study are available in the supplementary material of this article.
