# Supplementary material for: Tuning Self‐Assembly of Hole‐Selective Monolayers for Reproducible Perovskite/Silicon Tandem Solar Cells
Source: Small Methods. 2025 Feb 25;9(7):2401758. doi: 10.1002/smtd.202401758 (PMC12285630; doi:10.1002/smtd.202401758)
Supplement: Supplementary file 1 — Supporting Information [file SMTD-9-2401758-s001.docx]

Tuning Self-Assembly of Hole-Selective Monolayers for Reproducible Perovskite/Silicon Tandem Solar Cells

Oussama Er-raji^1,2,^*^,#^, Stefan Lange^3,*^, Carl Eric Hartwig^3^, Adi Prasetio^4^, Martin Bivour^1^, Martin Hermle^1^, Marko Turek^3^, Stefaan De Wolf^4^, Stefan W. Glunz^1,2^, Juliane Borchert^1,2^, Patricia S. C. Schulze^1^

** Equal contribution*

*# Corresponding author*

1 Fraunhofer Institute for Solar Energy Systems ISE, Heidenhofstr. 2, 79110 Freiburg, Germany

2 Chair of Photovoltaic Energy Conversion, Department of Sustainable Systems Engineering (INATECH), University of Freiburg, Emmy-Noether-Str.2, 79110 Freiburg, Germany

3 Fraunhofer Center for Silicon Photovoltaics CSP, Otto-Eissfeldt-Str. 12, 06120 Halle, Germany

4 KAUST Solar Center (KSC), Physical Sciences and Engineering Division (PSE), King Abdullah University of Science and Technology (KAUST), Thuwal 23955, Saudi Arabia


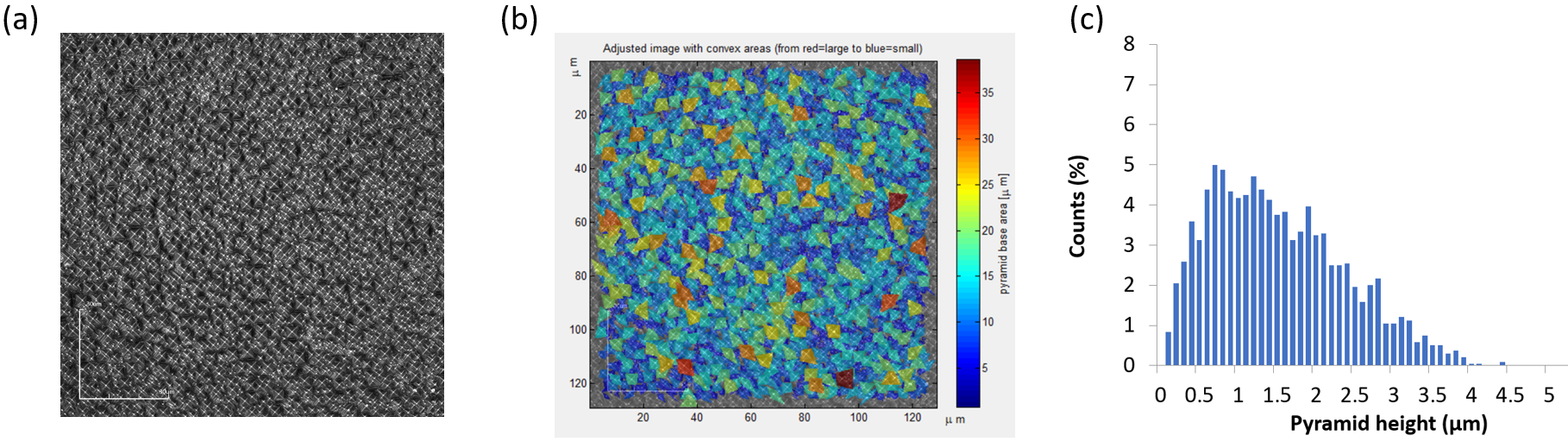


**Figure S1. LEXT optical microscope characterization of the front silicon texture pattern.** a) LEXT microscope image of a textured silicon bottom cell with random pyramid texture. b) optical and c) pyramid size distribution extracted from the LEXT image. Taken from ^1^ with permission.


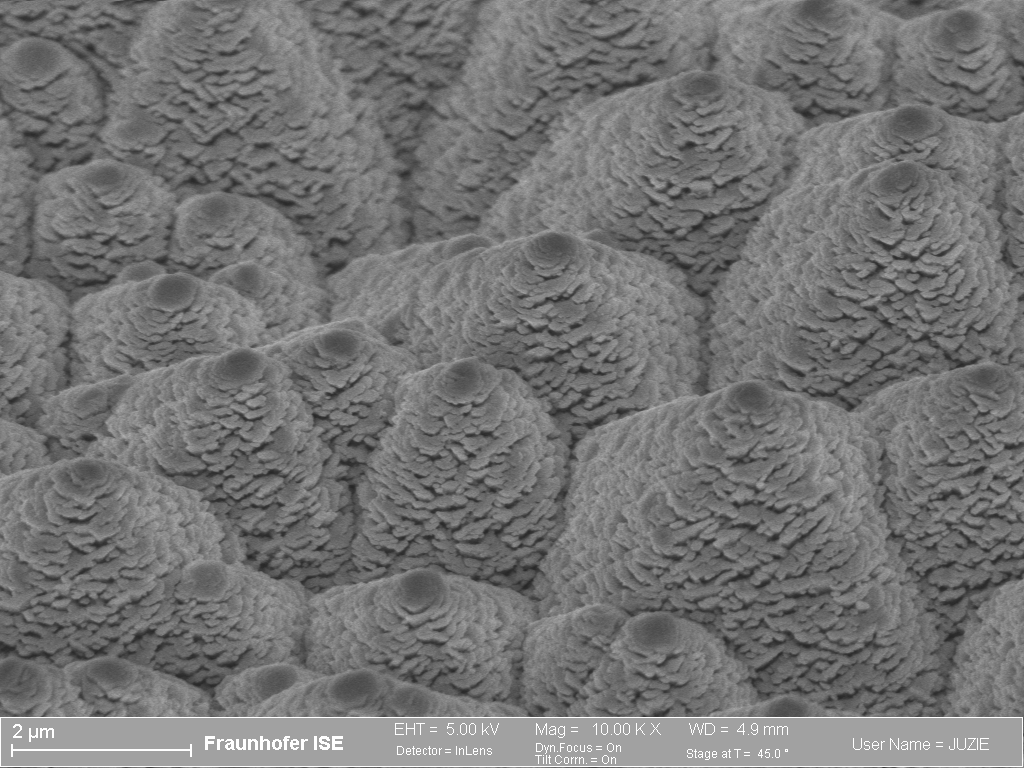

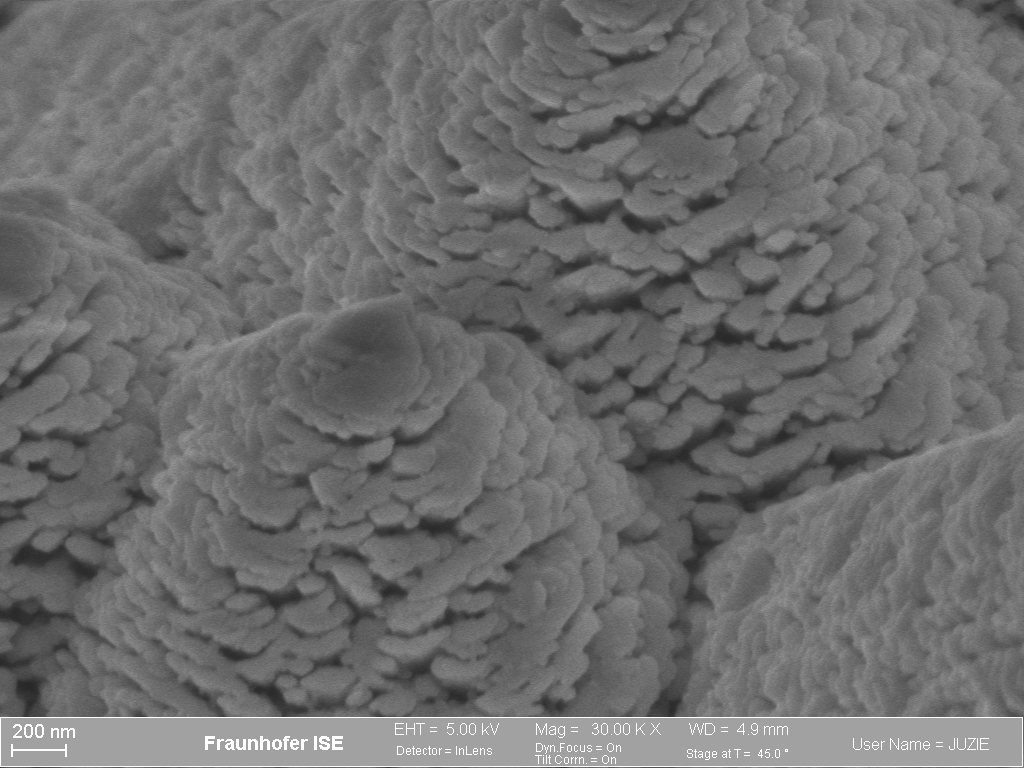


(b)

(a)


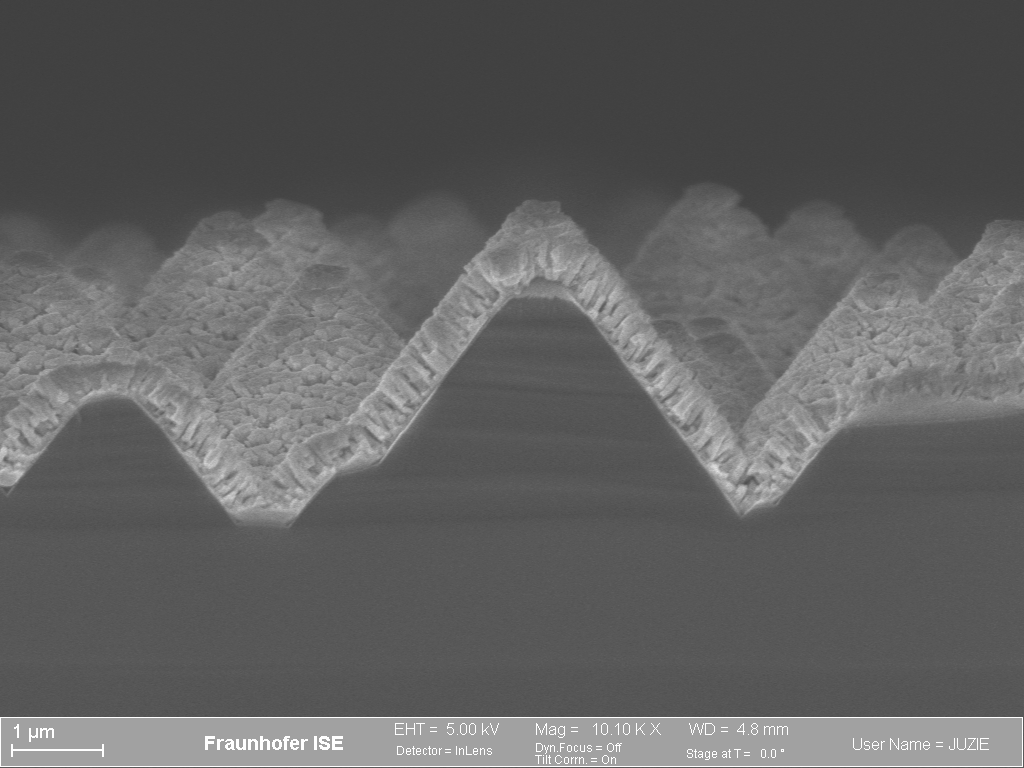

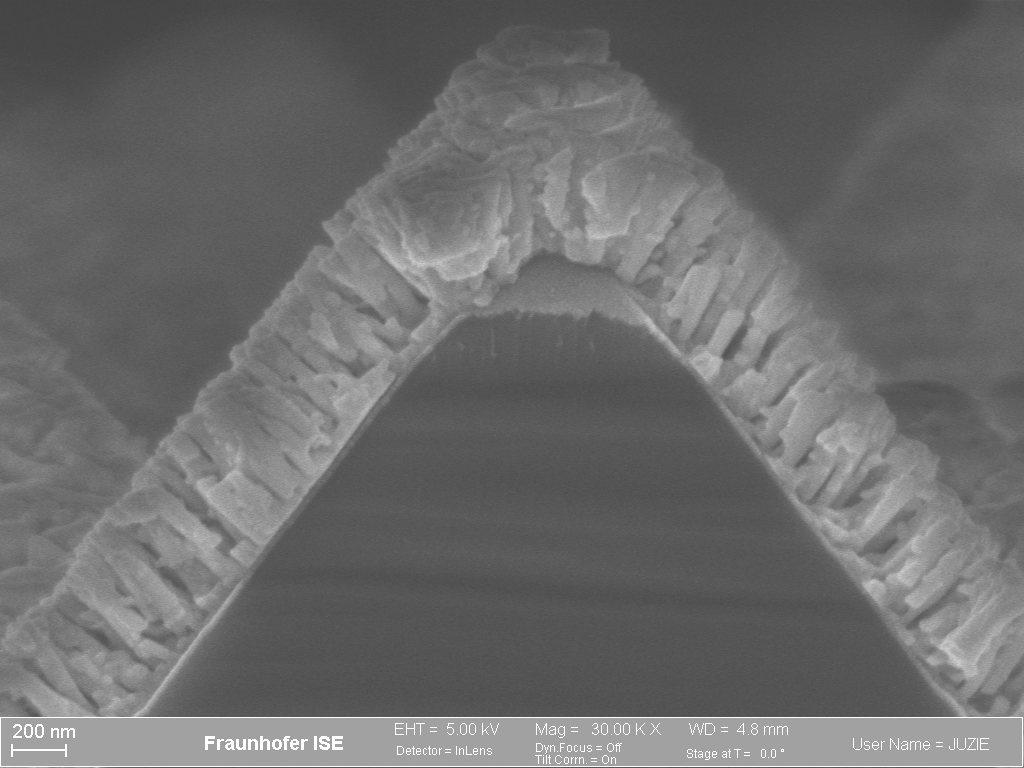


(d)

(c)

**Figure S2: Inorganic PbI_2_/CsI scaffold formation on textured silicon**. a,b) Top-view and c,d) cross-sectional scanning electron microscopy images of the evaporated inorganic template on top of textured silicon/ITO/2PACz. A porous film with high conformality can be seen.

(a)

(b)

**Figure S3: Impact of 2PACz annealing temperature on the final layer’s atomic composition using textured substrates.** a) Carbon to indium (C/In) and b) Phosphorus to indium (P/In) ratio change as a function of annealing temperature carried out in-situ (in gray) and ex-situ (in green) using the sample stack textured Si/ITO/2PACz. Overall a decrease in 2PACz elements (C and P) can be observed with increased annealing temperature. This change is however smaller compared to flat substrates as shown in Fig. 4 in the main manuscript.

**Figure S4: PL peak position of textured silicon/ITO/2PACz/perovskite stacks with 2PACz annealed at different temperatures.** Compared to the PL signal's height, which exhibits significant deviation during the laser exposure period for stacks where 2PACz is annealed at 100°C, the PL peak position remains stable across all variations.

**Figure S5: Transient photoluminescence measurements of textured silicon/ITO/2PACz/perovskite stacks with 2PACz annealed at different temperatures.** With elevated annealing temperatures of 2PACz, a higher fitted charge carrier lifetime is obtained.

(a)

(b)

**Figure S6: Impact of 2PACz annealing temperature on the work function via Kelvin probe measurements.** a) Work function across a line (distance between consecutive points is 0.5 mm), and b) average work function values. Similar to results from KPFM measurements presented in Fig. 5d of the main text, the results from Kelvin probe measurements, taken with a larger resolution area, demonstrate that increasing the 2PACz annealing temperature deepens the work function of the glass/ITO/2PACz stacks.

(e)

(d)

(c)

(b)

(a)

**Figure S7: Impact of 2PACz annealing temperature on the position of the highest occupied energy level (HOMO) in comparison to the valence band maximum of the perovskite.** Photoemission spectroscopy measurements in air (PESA) of a) ITO substrate, and 2PACz layers on ITO substrates annealed at b) 100°C, c) 125°C, d) 150°C, and e) the perovskite absorber. With increasing annealing temperature (100°C to 150°C), the energetic alignment improves with the HTL’s HOMO level and the perovskite’s VBM.

(b)

(a)

Silicon

Perovskite

**Figure S8: Impact of 2PACz annealing temperature on the *EQE* and reflection of the perovskite silicon tandem solar cells.** a) Normalized *EQE* and b) reflection of the perovskite silicon tandem solar cells with different SAM annealing temperatures. No significant difference can be observed

**Figure S9: Stabilized efficiency from fixed voltage measurements of champion tandem solar cells.** The stabilized efficiency for the 100°C annealing temperature is 28.15%. The stabilized efficiency for the 125°C annealing temperature is 29.44%. The stabilized efficiency for the 150°C annealing temperature is 28.17%.

| (in Å) | **100°C** | **125°C** | **150°C** | **175°C** | **400°C** | **500°C** |
| --- | --- | --- | --- | --- | --- | --- |
| **Flat** | 46 | 34 | 15 | 12 | 10 | 7 |
| **Textured** | 11 | 11 | 10 | 11 | - | - |

**Table S1: Impact of 2PACz annealing temperature and substrate type (textured vs flat) on the final 2PACz layer thickness.** The substrates consist of either flat or textured silicon with a 20 nm ITO recombination layer and spincoated 2PACz. The measured thickness designates the overlayer thickness which is a combination of the 2PACz layer and additional organic surface contaminants, amounted to be ~ 7.7 Å as indicated in Fig. 2f. Flat substrates show a larger dependence on 2PACz annealing temperature in contrast to textured substrates.

References

(1) Er-raji, O.; Bett, A.; Lange, S.; Nagel, H.; Bivour, M.; Schultz-Wittmann, O.; Hagendorf, C.; Hermle, M.; Borchert, J.; Glunz, S.; Schulze, P. Towards Efficient and Industrially-Compatible Fully-Textured Perovskite Silicon Tandem Solar Cells: Controlled Process Parameters for Reliable Perovskite Formation. *Progress in Photovoltaics* **2023**, 1-14. DOI: 10.1002/pip.3770. Published Online: Dec. 27, 2023.
